# Phase Contrast Imaging Based Microbubble Monitoring of Radiofrequency Ablation: An *ex vivo* Study

**DOI:** 10.3389/fonc.2020.01709

**Published:** 2020-08-25

**Authors:** Wei Huang, Jian Lu, Rongbiao Tang, Zhiyuan Wu, Qingbing Wang, Xiaoyi Ding, Zhongmin Wang, Kemin Chen

**Affiliations:** ^1^Department of Radiology, Ruijin Hospital, School of Medicine, Shanghai Jiaotong University, Shanghai, China; ^2^Department of Radiology, Ruijin Hospital/Luwan Branch, School of Medicine, Shanghai Jiaotong University, Shanghai, China

**Keywords:** radiofrequency ablation, synchrotron, microbubbles, radiation, phase contrast imaging

## Abstract

**Background:**

To explore the potential of synchrotron radiation (SR) phase contrast imaging (PCI) for real-time microbubble formation monitoring during radiofrequency ablation (RFA).

**Methods:**

RFA was performed on *ex vivo* porcine muscle tissue using unipolar and multi-tined expandable electrodes. Images of microbubble formation in the samples were captured by both SR PCI and absorption contrast imaging. The synchronous ablation temperature was recorded. Each RFA electrode type group contained 6 samples. Ablation size was assessed by histologic examination.

**Results:**

Microbubble formation during RFA could be visualized by SR PCI. The diameter of the microbubbles revealed on the image ranged from tens of microns to several millimeters, and these microbubbles first appeared at the edge of the RFA electrode when the target region temperature reached approximately 60°C and rapidly extended outwards. The average microbubble range measured on PCI was 17.66 ± 0.74 mm. The average range of coagulation necrosis measured by histological examination was 17.22 ± 0.38 mm. There was no significant difference between them (*P* > 0.05). The range of microbubbles corresponded to the ablation zone.

**Conclusion:**

PCI enabled real-time high-resolution visualization of microbubble formation during RFA, indicating a potential for its use in ablation monitoring.

## Background

Radiofrequency ablation (RFA) has gained widespread acceptance in the local therapy of various benign and malignant solid lesions, particularly those involving the liver, lung, kidney and musculoskeletal system (1–8). It is usually performed percutaneously under image guidance and is appropriate for inoperable patients with comorbidities.

In clinical practice, it is difficult to predict the RFA zone. The size and shape of the RFA zone can vary according to many factors, including the location of the lesion, the histological characteristics of the lesion and adjacent tissues, the type of electrode, duration of ablation and the heat sink phenomenon (9–11). This disadvantage may explain why the local recurrence rate after RFA is higher than that after surgery, especially for voluminous lesions (12–16). Some studies show a sharp rise in the rate of local recurrences and a decline of overall survival for tumors over 3–5 cm (17–19). And nowadays, the indication of RFA is usually limited to the lesions less than 5 cm. If the RFA zone can be monitored accurately during the procedure, the relatively high local residue and recurrence rate could be overcome.

Currently, RFA procedures are generally performed under ultrasound (US), computed tomography (CT), or magnetic resonance imaging (MRI) guidance (20–27). These traditional image guidance methods can help to localize the RFA electrode to the lesion site but do not serve as a real-time monitoring tool to evaluate the ablation zone during the procedure due to their physical limitations. To overcome these disadvantages, a new monitoring method should be employed. Synchrotron radiation (SR) phase contrast imaging (PCI) is more functional than conditional absorption contrast imaging (ACI) and is characterized by its high spatial resolution of sub-micron level and high temporal resolution of milliseconds (28–30). Moreover, according to the phase shift caused by variations in the refractive index and thickness of materials (31), PCI is effective at the border of two different structures and can provide significant edge enhancement for low-density materials, including gas, organic materials, and soft tissues (29, 32–34).

In previous studies, the range of microbubbles formed during RFA corresponded to the RFA zone (35, 36). Therefore, in this study, we planned to use PCI to reveal microbubble formation during RFA using two different types of frequently used RFA electrodes and evaluate the potential for real-time monitoring of RFA.

## Materials and Methods

All experiments were conducted in accordance with the guidelines established and approved by Shanghai Jiao Tong University’s Institutional Animal Care and Use Committee (Approval number: B-2015-011).

### Sample Preparation

Porcine muscle (Lingchang Biotech, Shanghai, China) was used and cut into appropriately sized samples (3 × 3 × 3 cm for the multi-tined electrode, 2.5 × 2.5 × 2.5 cm for the uni-polar electrode).

In clinical practice, RFA is most frequently applied to liver and muscular like tissue. Porcine liver was excluded at this study, because *ex vivo* hepatic vessels contain gas, which can be revealed by PCI and hamper the observation of micro-bubble formation during RFA.

### SR Parameters

The study was performed in the BL13W1 beamline at the Shanghai Synchrotron Radiation Facility (SSRF, China). A 3.5 GeV electron storage ring generated X-rays with an energy range of 8–72.5 keV and an average beam current of 180 mA. X-rays were then monochromatized by a double-crystal monochromator with Si (111) and Si (311) crystals at an energy level of 22 KeV. Energy resolution was ΔE/E<5 × 10^3^. Incoming X-rays were converted into visible light using a 100 μm thick CdWO4 cleaved single crystal scintillator and consecutively captured by a CCD camera (Photonic Science, Britain) with pixel size of 9 μm. The sample was placed 34 m downstream of the SR source. The distance between the sample and the CCD camera had a changeable range of 8 m.

### ACI and PCI Parameters

A total of 0.2 ml of gas was injected into the central of one 2.5 × 2.5 × 2.5 cm sample by a 17G needle, in order to identify the different presentation of bubbles on SR ACI and PCI.

**FIGURE 1:**
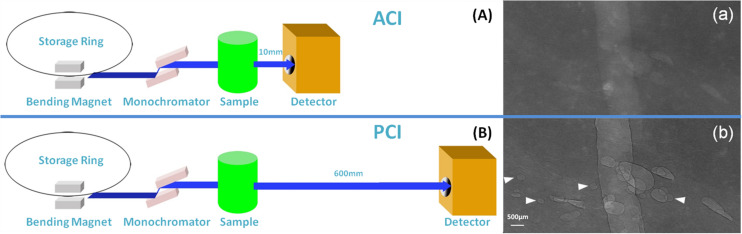
Schematic of SR ACI and PCI, and the images of the bubbles. SR images of the same sample were obtained at 22 KeV with two different object-to-detector distances of 10 and 600 mm. At a distance of 10 mm, ACI was obtained **(A)**; at a distance of 600 mm, PCI was captured **(B)**. Bubbles could be clearly depicted by PCI and displayed good contrast with the peripheral tissue, especially the sharp edges **(b)**. The contrast between bubbles and surrounding tissues was not clear in ACI **(a)**. Arrows indicate bubbles visible by PCI but invisible by ACI. The pixel size was 9 μm × 9 μm, and the exposure time was 40 ms.

Synchrotron radiation images of this sample were obtained at 22 KeV with two different object-to-detector distances of 10 and 600 mm. At an object-to-detector distance of 10 mm, detector was placed immediately behind the sample, the phase contrast effect is minimal, ACI was obtained (Figure 1A). The phase contrast effect increases with the augmentation of object-to-detector distance. At an object-to-detector distance of 600 mm, PCI can be captured (Figure 1B). As the quality of visible bubbles using PCI was greater than that using ACI. PCI was then used to monitor the following RFA procedure.

### RFA Parameters

Radiofrequency ablation was performed with a RITA Model 1500X radiofrequency generator, 250 W, 460 Hz frequency (RITA Medical Systems, Mountain View, CA, United States). The RFA electrodes included a unipolar electrode and multi-tined expandable electrodes. The RFA time was 7 min for the unipolar electrode and 5 min for the multi-tined expandable electrodes with a 1 cm expandable range. The energy level was 90 W. The target goal temperature was set to 105°C, and the real-time actual temperature was measured by thermodetectors in the electrodes. The RFA electrodes were localized to the center of the sample, which was placed on a translation stage. The electrode axis was perpendicular to the synchrotron source and confirmed by SR fluoroscopy. The loop electrode was adhered to the underside of the sample. Each RFA electrode type group contained six samples, and there were 12 RFA ablations performed during the course of this study.

### Image Acquisition

Images were captured with an exposure time of 40 ms at 98 frames per minute. Background noise was eliminated using Image-Pro Plus 6.0 (Media Cybernetics Inc., United States), and image quality was compared. The microbubble formation process was pseudo-colored using a normalization algorithm via MATLAB 7.0 (MathWorks, United States). The range of microbubbles, RFA temperature and RFA time were synchronously recorded.

### Pathological Analysis

Samples were bisected across the long axe of the electrode track for immediate gross examination. The width of the RFA ablation zone was measured by calipers. The sample was then fixed in 10% formalin and later stained with hematoxylin and eosin (H&E) for histomorphological analysis.

### Statistical Analysis

The data are presented as the means ± standard error. Using SPSS 19.0 software, the range of microbubble measured on the PCI and the range of RFA zone showed on gross examination and histomorphological analysis were compared using the paired *t*-test. A *P*-value of less than 0.05 was defined as statistically significant.

## Results

### Comparison of Bubble Morphology Between PCI and ACI

The PCI and ACI characteristics of bubbles are displayed in Figure 1 after an injection of gas in the same sample. The contrast between bubbles and surrounding tissue was not clear in ACI (Figure 1a). After adjusting the sample-to-detector distance to 600 mm, bubbles with sharp edges could be clearly visualized by PCI (Figure 1b). The quality of visible bubbles using PCI was greater than that using ACI.

### PCI of Microbubble Formation During RFA

**FIGURE 2:**
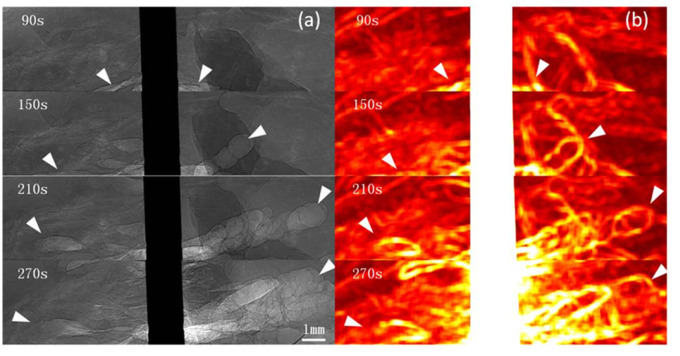
PCI of microbubble diffusion process during unipolar electrode RFA. PCI of the microbubble diffusion process during RFA using a unipolar electrode **(a)** and pseudo-colored with a normalization algorithm **(b)**. Microbubbles were revealed 40 s after ablation and distributed at the edge of the electrode and adjacent tissues. From the electrode surface outward, microbubbles continued to increase and expand rapidly. The density and range of microbubbles increased with the rise in temperature and RFA duration **(a)**. The range of microbubbles is highlighted on the pseudo-colored image **(b)**. Arrowheads indicate microbubble ranges. The pixel size was 9 μm × 9 μm, and the exposure time was 40 ms.

**FIGURE 3:**
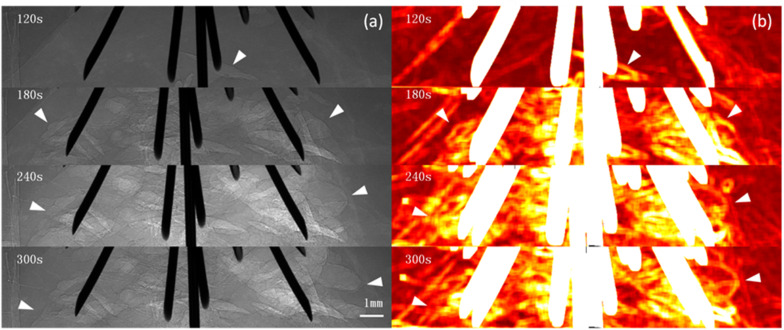
PCI of microbubble diffusion process during multi-tined expandable electrodes RFA. PCI of the microbubble diffusion process during RFA using multi-tined expandable electrodes **(a)** and pseudo-colored with a normalization algorithm **(b)**. Microbubbles first appeared at the edge of electrodes 60 s after ablation when the temperature reached 60°C **(a)**. Cleft-like microbubbles; microbubbles continued to increase and expand rapidly outward. The distribution of microbubbles could be monitored in real time by PCI **(a)**. The highlighted area corresponding to the microbubble range first appeared at the edge of the electrodes and then spread into surrounding tissues **(b)**. Arrowheads indicate microbubble ranges. The pixel size was 9 μm × 9 μm, and the exposure time was 40 ms.

Images were obtained 20 s after RFA began. For the unipolar electrode, 40 s after RFA began, when the local temperature reached approximately 60°C, microbubbles began to appear at the electrode edge. For the multi-tined expandable electrodes, 60 s after RFA began, microbubbles began to appear at the same temperature of 60°C. During RFA, from the electrode surface outward, the newly generated microbubbles were irregular and cleft-like in shape. As the temperature rose, microbubbles continued to increase and expand rapidly outward. The density and range of microbubbles increased with the rise in temperature and RFA duration. The complete microbubble diffusion process was revealed and pseudo-colored (Figures 2, 3).

**FIGURE 4:**
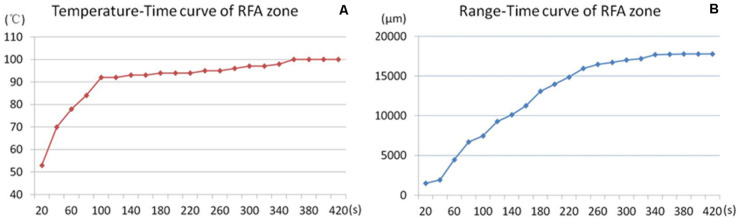
Temperature-range-time curve of RFA. RFA temperature-time curve **(A)** and microbubbles range–time curve **(B)** of the unipolar electrode during RFA. The RFA temperature rose rapidly to 90°C in the first 100 s and was maintained at approximately 100°C for the remaining ablation time **(A)**. The microbubble range increased rapidly in the first 4 min, and the increasing slope declined in the last 3 min.

The RFA temperature-time curve and microbubble range-time curve of the unipolar electrode were recorded (Figure 4).

### PCI of Microbubble Transformation After RFA

**FIGURE 5:**
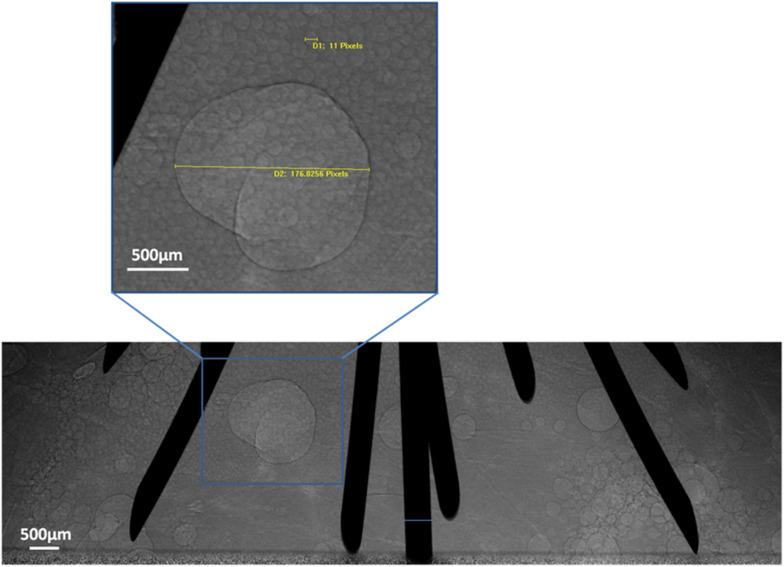
Microbubble transformation tendency. PCI of microbubble transformation 30 min after RFA using multi-tined expandable electrodes. Most microbubbles were tens of microns in size. Some fused together to form larger bubbles. One bubble was magnified and measured in the magnified images of the region.

Images were captured from 10 to 30 min after RFA. Microbubbles remained in the tissue. Gradually, the microbubbles became regular circle- or oval-shaped and displayed a tendency to fuse together. The diameter of the microbubbles ranged from tens of microns to several millimeters (Figure 5).

### Tissue Section Images After RFA

**FIGURE 6:**
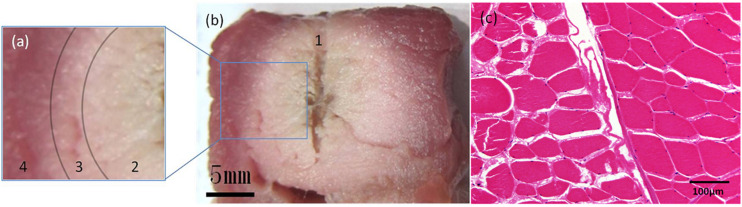
Pathological examinations. Sectional images and H&E-stained image of the sample after RFA using a unipolar electrode. On sectional images **(a,b)**, the sample could be divided into 4 parts: 1. The central fissure is the trace of the unipolar electrode; 2. the adjacent white region indicates complete coagulation necrosis; 3. the pink transitional region indicates partial coagulation necrosis; and 4. the peripheral red region depicts intact tissue. The transition between parts 2, 3, and 4 is gradual. On the H&E-stained image **(c)**, the ablation zone is on the left half. Muscle bundles fissure after RFA, and their interval is enlarged. Cytoplasm of muscle cells appears pink, and the nuclei are dark and faint.

Sectional images of the sample and pathological sections (Figure 6) were obtained after RFA.

Radiofrequency ablation was performed on six samples using a unipolar electrode. The average microbubble range measured on PCI was 17.66 ± 0.74 mm. The average range of coagulation necrosis (including complete and partial coagulation necrotic areas) measured by histological examination was 17.22 ± 0.38 mm. There was no significant difference between them (*P* > 0.05).

The diameter of the ablation zone of the multi-tined expandable electrodes was more than 20 mm. The final microbubble range could not be measured because the field of our CCD vision was limited to 20 mm.

## Discussion

Radiofrequency ablation electrodes can deliver a high-frequency sinusoidal electromagnetic current that induces agitation of tissue ions, after which energy dissipates as heat through ionic friction (37). If the tissue temperature reaches approximately 60°C and this temperature is maintained for a few seconds, irreversible damage can occur due to DNA denaturation, manifesting as coagulation necrosis (9).

The key point of real-time monitoring of RFA is to identify coagulation necrosis from the adjacent tissue. However, the problem in exact quantification of the coagulation necrotic zone still prevails and hinders the treatment effect of RFA therapy (38). The imaging techniques used during the RFA procedure include US, CT, and MRI, which serve as ideal guidance tools. However, they have some limitations in the real-time monitoring of the ablation area. Hypersonic microbubble formation during the ablation and acoustic shadow of the RFA electrode shield the lesion and impair the quality of US images (35, 39). CT cannot clearly delineate the boundary between the coagulation zone and the area of peripheral edema because of their similar densities. Frequency interference between the RF generator and the MR imager is difficult to avoid. Most current MRI and RFA devices do not allow for simultaneous MRI and the application of RFA energy (25).

Previous studies have used US and US Nakagami imaging to monitor RFA microbubble formation, the range of which corresponded with the ablation zone (35, 36). However, microbubbles of tens of microns in diameter cannot be directly visualized using US imaging, as it has limited spatial resolution. Moreover, the posterior margin of the ablation zone is difficult to observe due to electrode acoustic shadow and irregular reflections of ultrasound by microbubbles (35).

Variations in the refractive index between gas and soft tissue are evident and can cause an edge enhancement effect on PCI. This effect is a significant advantage of PCI and is the result of the high spatial coherence of inline PCI achievable with a large object-to-detector distance (32). PCI is more suitable for microbubble observation than ACI and can reveal microbubbles of tens of microns in size. With the characteristic of high temporal resolution, PCI can even detect microbubbles newly formed on the surface of RFA electrodes. In our study, microbubble formation during the entire RFA procedure and the transformation after RFA could be directly visualized by PCI.

The hypothesis of microbubble formation during RFA is as follows. First, when the temperature of the tissue reaches the boiling point, vapor is generated in the form of microbubbles. Second, microbubbles in the blood within microvessels flow out because of the hyperemia induced by ablation (40). As the samples used in this study were *ex vivo*, the potential interference of microvessels could be excluded, and the first hypothesis might be more reasonable.

Microbubbles appeared when the temperature increased and diffused outward from the surface of RFA electrodes. We observed a phenomenon during this procedure: the temperature threshold of microbubble formation was 60°C, which is much lower than the boiling point of water. There are two possible explanations: First, because of the relatively low heat conductivity of the tissue, vapor could be generated at 60°C and manifest as microbubbles. Second, local temperature may have reached the boiling point, but the area may be limited and could not be accurately measured by thermodetectors in the electrodes. The relationship between microbubble formation and the exact local temperature should be explored in future studies.

Nevertheless, microbubble formation indicates that the local tissue temperature has reached at least 60°C. At this temperature, coagulation necrosis in tissues is irreversible (9). According to this phenomenon, the microbubble range revealed by PCI may reflect the coagulation necrotic area.

After RFA, microbubbles remained in the ablation zone on PCI. Some fused together and formed bubbles of several millimeters in diameter. This phenomenon can explain the origin of bubbles observed on CT images in some clinical cases after RFA (9).

In previous studies, SR PCI has showed some advantages in the clinical imaging diagnostic domain. Compared to the traditional mammography, the image quality was improved by using PCI (41, 42). A significant reduction of radiation dose was obtained by SR high resolution CT (43). According to our study, with its high resolution of low-density materials and high temporal resolution, SR PCI has the potential to be used in real-time monitoring of the RFA zone via microbubble imaging.

There are some limitations to our study. First, an *ex vivo* study might not reflect actual microbubble transformation *in vivo*, and the influence of microvessel perfusion and the heat sink phenomenon should be considered. Second, the RFA zone of multi-tined expandable electrodes was larger than the field of the CCD, and its range could not be measured by PCI. Third, although in our study, the range of microbubbles after RFA using unipolar electrodes was close to that measured on histological cross-sections, which contained complete and partial coagulation necrotic areas; additionally, the transition between complete coagulation necrosis and partial coagulation necrosis was gradual, and the boundary was indistinct. Further quantitative studies should be carried out to analyze the relationship between the density of microbubbles and the degree of coagulation necrosis.

## Conclusion

Synchrotron radiation PCI enabled real-time high-resolution visualization of microbubble formation during RFA. The temperature threshold of micro-bubble appearance was about 60°C. At this temperature level, protein denaturation and cellular damage is irreversible, meaning that the range of micro-bubbles corresponds with cell necrosis area, which was confirmed by pathological examinations in our study. In summary, SR PCI has a potential for real-time monitoring of RFA zone.

## Data Availability Statement

The datasets generated for this study are available on request to the corresponding author.

## Ethics Statement

The animal study was reviewed and approved by the Shanghai Jiao Tong University School of Medicine Institutional Animal Care & Use Committee.

## Author Contributions

WH, JL, and ZWa designed the research and supervised the report. ZWu and RT performed the research. WH and JL wrote the manuscript. XD, KC, and ZWa supervised the report. KC, ZWu, and QW analyzed the data. All authors contributed to the article and approved the submitted version.

## Conflict of Interest

The authors declare that the research was conducted in the absence of any commercial or financial relationships that could be construed as a potential conflict of interest.

## Abbreviations

ACI: absorption contrast imaging
CT: computed tomography
MRI: magnetic resonance imaging
PCI: phase contrast imaging
RFA: radiofrequency ablation
SR: synchrotron radiation
US: ultrasound.

## References

[1] Kim YS Lim HK Rhim H Lee MW Choi D Lee WJ Ten-year outcomes of percutaneous radiofrequency ablation as first-line therapy of early hepatocellular carcinoma: analysis of prognostic factors. *J Hepatol.* (2013) 58:89–97. 10.1016/j.jhep.2012.09.020 23023009

[2] Stoltz A Gagniere J Dupre A Rivoire M. Radiofrequency ablation for colorectal liver metastases. *J Visc Surg.* (2014) 151(Suppl. 1):S33–44. 10.1016/j.jviscsurg.2013.12.005 24582728

[3] Lanuti M Sharma A Digumarthy SR Wright CD Donahue DM Wain JC Radiofrequency ablation for treatment of medically inoperable stage I non-small cell lung cancer. *J Thorac Cardiovasc Surg.* (2009) 137:160–6. 19154919 10.1016/j.jtcvs.2008.08.034

[4] Lyons NJ Pathak S Daniels IR Spiers A Smart NJ. Percutaneous management of pulmonary metastases arising from colorectal cancer; a systematic review. *Eur J Surg Oncol.* (2015) 41:1447–55. 26358568 10.1016/j.ejso.2015.07.018

[5] Breen DJ Rutherford EE Stedman B Roy-Choudhury SH Cast JE Hayes MC Management of renal tumors by image-guided radiofrequency ablation: experience in 105 tumors. *Cardiovasc Intervent Radiol.* (2007) 30:936–42. 17573550 10.1007/s00270-007-9090-x PMC2700242

[6] Gervais DA McGovern FJ Arellano RS McDougal WS Mueller PR. Radiofrequency ablation of renal cell carcinoma: part 1, Indications, results, and role in patient management over a 6-year period and ablation of 100 tumors. *AJR Am J Roentgenol.* (2005) 185:64–71. 10.2214/ajr.185.1.01850064 15972400

[7] Botsa E Mylona S Koutsogiannis I Koundouraki A Thanos L. CT image guided thermal ablation techniques for palliation of painful bone metastases. *Ann Palliat Med.* (2014) 3:47–53. 25841503 10.3978/j.issn.2224-5820.2014.04.02

[8] Motamedi D Learch TJ Ishimitsu DN Motamedi K Katz MD Brien EW Thermal ablation of osteoid osteoma: overview and step-by-step guide. *Radiographics.* (2009) 29:2127–41. 19926767 10.1148/rg.297095081

[9] Kim YS Rhim H Lim HK Choi D Lee MW Park MJ. Coagulation necrosis induced by radiofrequency ablation in the liver: histopathologic and radiologic review of usual to extremely rare changes. *Radiographics.* (2011) 31:377–90. 21415185 10.1148/rg.312105056

[10] dos Santos I Haemmerich D Pinheiro Cda S da Rocha AF. Effect of variable heat transfer coefficient on tissue temperature next to a large vessel during radiofrequency tumor ablation. *Biomed Eng Online.* (2008) 7:21. 10.1186/1475-925x-7-21 18620566 PMC2500024

[11] Pillai K Akhter J Chua TC Shehata M Alzahrani N Al-Alem I Heat sink effect on tumor ablation characteristics as observed in monopolar radiofrequency, bipolar radiofrequency, and microwave, using ex vivo calf liver model. *Medicine.* (2015) 94:e580. 10.1097/md.0000000000000580 25738477 PMC4553952

[12] Ito T Tanaka S Iwai S Takemura S Hagihara A Uchida-Kobayashi S Outcomes of laparoscopic hepatic resection versus percutaneous radiofrequency ablation for hepatocellular carcinoma located at the liver surface: a case-controlled study with propensity score matching. *Hepatol Res.* (2015) 46:565–74. 10.1111/hepr.12592 26386248

[13] Han K Ko HK Kim KW Won HJ Shin YM Kim PN. Radiofrequency ablation in the treatment of unresectable intrahepatic cholangiocarcinoma: systematic review and meta-analysis. *J Vasc Interv Radiol.* (2015) 26:943–8. 25899049 10.1016/j.jvir.2015.02.024

[14] Weis S Franke A Mossner J Jakobsen JC Schoppmeyer K. Radiofrequency (thermal) ablation versus no intervention or other interventions for hepatocellular carcinoma. *Cochrane Database Syst Rev.* (2013) 12:CD003046. 10.1002/14651858.CD003046.pub3 24357457 PMC11931681

[15] Wang Y Luo Q Li Y Deng S Wei S Li X. Radiofrequency ablation versus hepatic resection for small hepatocellular carcinomas: a meta-analysis of randomized and nonrandomized controlled trials. *PLoS One.* (2014) 9:e84484. 10.1371/journal.pone.0084484 24404166 PMC3880302

[16] Hasegawa K Kokudo N Makuuchi M Izumi N Ichida T Kudo M Comparison of resection and ablation for hepatocellular carcinoma: a cohort study based on a Japanese nationwide survey. *J Hepatol.* (2013) 58:724–9. 23178708 10.1016/j.jhep.2012.11.009

[17] Seror O N’Kontchou G Ibraheem M Ajavon Y Barrucand C Ganne N Large (>or=5.0-cm) HCCs: multipolar RF ablation with three internally cooled bipolar electrodes–initial experience in 26 patients. *Radiology.* (2008) 248:288–96. 10.1148/radiol.2481071101 18483229

[18] Morgan JH III Royer GM Hackett P Gamblin TC McCampbell BL Conforti A Radio-frequency ablation of large, nonresectable hepatic tumors. *Am Surg.* (2004) 70:1035–8. 15663040

[19] Mulier S Ni Y Jamart J Ruers T Marchal G Michel L. Local recurrence after hepatic radiofrequency coagulation: multivariate meta-analysis and review of contributing factors. *Ann Surg.* (2005) 242:158–71. 10.1097/01.sla.0000171032.99149.fe 16041205 PMC1357720

[20] Rajesh S Mukund A Arora A Jain D Sarin SK. Contrast-enhanced US-guided radiofrequency ablation of hepatocellular carcinoma. *J Vasc Interv Radiol.* (2013) 24:1235–40. 23796857 10.1016/j.jvir.2013.04.013

[21] Solbiati L Ierace T Tonolini M Cova L. Guidance and monitoring of radiofrequency liver tumor ablation with contrast-enhanced ultrasound. *Eur J Radiol.* (2004) 51(Suppl.):S19–23. 10.1016/j.ejrad.2004.03.035 15311434

[22] Iguchi T Hiraki T Gobara H Fujiwara H Matsui Y Soh J Percutaneous radiofrequency ablation of lung cancer presenting as ground-glass opacity. *Cardiovasc Intervent Radiol.* (2015) 38:409–15. 24938905 10.1007/s00270-014-0926-x

[23] Sommer CM Lemm G Hohenstein E Bellemann N Stampfl U Goezen AS CT-guided bipolar and multipolar radiofrequency ablation (RF ablation) of renal cell carcinoma: specific technical aspects and clinical results. *Cardiovasc Intervent Radiol.* (2013) 36:731–7. 10.1007/s00270-012-0468-z 22926302

[24] Takaki H Yamakado K Nakatsuka A Yamada T Shiraki K Takei Y Frequency of and risk factors for complications after liver radiofrequency ablation under CT fluoroscopic guidance in 1500 sessions: single-center experience. *AJR Am J Roentgenol.* (2013) 200:658–64. 10.2214/AJR.12.8691 23436859

[25] Clasen S Boss A Schmidt D Schraml C Fritz J Schick F MR-guided radiofrequency ablation in a 0.2-T open MR system: technical success and technique effectiveness in 100 liver tumors. *J Magn Reson Imaging.* (2007) 26:1043–52. 10.1002/jmri.21120 17896364

[26] Fischbach F Lohfink K Gaffke G Wybranski C Mohnike K Wonneberger U Magnetic resonance-guided freehand radiofrequency ablation of malignant liver lesions: a new simplified and time-efficient approach using an interactive open magnetic resonance scan platform and hepatocyte-specific contrast agent. *Invest Radiol.* (2013) 48:422–8. 10.1097/RLI.0b013e3182803dae 23399808

[27] Clasen S Rempp H Hoffmann R Graf H Pereira PL Claussen CD. Image-guided radiofrequency ablation of hepatocellular carcinoma (HCC): is MR guidance more effective than CT guidance? *Eur J Radiol.* (2014) 83:111–6. 24161781 10.1016/j.ejrad.2013.09.018

[28] Nuzzo S Peyrin F Cloetens P Baruchel J Boivin G. Quantification of the degree of mineralization of bone in three dimensions using synchrotron radiation microtomography. *Med Phys.* (2002) 29:2672–81. 10.1118/1.1513161 12462734

[29] Tang R Huang W Yan F Lu Y Chai WM Yang GY In-line phase contrast imaging of hepatic portal vein embolization with radiolucent embolic agents in mice: a preliminary study. *PLoS One.* (2013) 8:e80919. 10.1371/journal.pone.0080919 24324646 PMC3851775

[30] Lewis RA. Medical phase contrast x-ray imaging: current status and future prospects. *Phys Med Biol.* (2004) 49:3573–83. 10.1088/0031-9155/49/16/005 15446788

[31] Spanne P Raven C Snigireva I Snigirev A. In-line holography and phase-contrast microtomography with high energy x-rays. *Phys Med Biol.* (1999) 44:741–9. 10.1088/0031-9155/44/3/016 10211807

[32] Olubamiji AD Izadifar Z Chen DX. Synchrotron imaging techniques for bone and cartilage tissue engineering: potential, current trends, and future directions. *Tissue Eng Part B Rev.* (2014) 20:503–22. 10.1089/ten.teb.2013.0493 24517187

[33] Tang R Li WX Huang W Yan F Chai WM Yang GY CO(2)-based in-line phase contrast imaging of small intestine in mice. *Sci Rep.* (2013) 3:2313. 10.1038/srep02313 PMC3727055 23896957

[34] Pisano ED Johnston RE Chapman D Geradts J Iacocca MV Livasy CA Human breast cancer specimens: diffraction-enhanced imaging with histologic correlation–improved conspicuity of lesion detail compared with digital radiography. *Radiology.* (2000) 214:895–901. 10.1148/radiology.214.3.r00mr26895 10715065

[35] Nouso K Shiraga K Uematsu S Okamoto R Harada R Takayama S Prediction of the ablated area by the spread of microbubbles during radiofrequency ablation of hepatocellular carcinoma. *Liver Int.* (2005) 25:967–72. 16162154 10.1111/j.1478-3231.2005.01145.x

[36] Zhou Z Wu S Wang CY Ma HY Lin CC Tsui PH. Monitoring radiofrequency ablation using real-time ultrasound Nakagami imaging combined with frequency and temporal compounding techniques. *PLoS One.* (2015) 10:e0118030. 10.1371/journal.pone.0118030 25658424 PMC4320093

[37] Tatli S Tapan U Morrison PR Silverman SG. Radiofrequency ablation: technique and clinical applications. *Diagn Interv Radiol.* (2012) 18:508–16. 10.4261/1305-3825.DIR.5168-11.1 22407695

[38] Singh S Repaka R. Numerical study to establish relationship between coagulation volume and target tip temperature during temperature-controlled radiofrequency ablation. *Electromagn Biol Med.* (2018) 37:13–22. 10.1080/15368378.2017.1422262 29308914

[39] Chiou SY Liu JB Needleman L. Current status of sonographically guided radiofrequency ablation techniques. *J Ultrasound Med.* (2007) 26:487–99. 10.7863/jum.2007.26.4.487 17384046

[40] McGhana JP Dodd GD III. Radiofrequency ablation of the liver: current status. *AJR Am J Roentgenol.* (2001) 176:3–16. 10.2214/ajr.176.1.1760003 11133529

[41] Castelli E Tonutti M Arfelli F Longo R Quaia E Rigon L Mammography with synchrotron radiation: first clinical experience with phase-detection technique. *Radiology.* (2011) 259:684–94. 21436089 10.1148/radiol.11100745

[42] Longo R Tonutti M Rigon L Arfelli F Dreossi D Quai E Clinical study in phase- contrast mammography: image-quality analysis. *Philos Trans A Math Phys Eng Sci.* (2014) 372:20130025. 10.1098/rsta.2013.0025 24470410

[43] Labriet H Nemoz C Renier M Berkvens P Brochard T Cassagne R Significant dose reduction using synchrotron radiation computed tomography: first clinical case and application to high resolution CT exams. *Sci Rep.* (2018) 8:12491. 10.1038/s41598-018-30902-y PMC6104060 30131501

